# A rare subclinical or mild type of Becker muscular dystrophy caused by a single exon 48 deletion of the dystrophin gene

**DOI:** 10.1007/s13353-017-0391-8

**Published:** 2017-02-28

**Authors:** Janusz G. Zimowski, Jacek Pilch, Magdalena Pawelec, Joanna K. Purzycka, Jolanta Kubalska, Karolina Ziora-Jakutowicz, Magdalena Dudzińska, Jacek Zaremba

**Affiliations:** 1 Department of Genetics, Institute of Psychiatry and Neurology, ul. Sobieskiego 9, 02-957 Warszawa, Poland; 2 Department of Pediatrics and Neurology for Children and Adolescents, Medical University of Silesia, ul. Medyków 16, 40-752 Katowice, Poland; 3 Children’s Neurology Ward, Dr. E. Hanke Centre of Pediatrics and Oncology of Chorzów, ul. Truchana 7, 41-500 Chorzów, Poland

**Keywords:** Asymptomatic mutations, Dystrophin gene, Dystrophinopathy, Exon 48 deletion, Subclinical BMD

## Abstract

In the material of 227 families with Becker muscular dystrophy (BMD), we found nine non-consanguineous families with 17 male individuals carrying a rare mutation—a single exon 48 deletion of the dystrophin gene—who were affected with a very mild or subclinical form of BMD. They were usually detected thanks to accidental findings of elevated serum creatine phosphokinase (sCPK). A thorough clinical analysis of the carriers, both children (12) and adults (5), revealed in some of them muscle hypotonia (10/17) and/or very mild muscle weakness (9/17), as well as decreased tendon reflexes (6/17). Adults, apart from very mild muscle weakness and calf hypertrophy in some, had no significant abnormalities on neurological assessments and had good exercise tolerance. Parents of the children carriers of the exon 48 deletion are usually unaware of their children being affected, and possibly at risk of developing life-threatening cardiomyopathy. The same concerns the adult male carriers. Therefore, the authors postulate undertaking preventive measures such as cascade screening of the relatives of the probands. Newborn screening programmes of Duchenne muscular dystrophy (DMD)/BMD based on sCPK marked increase may be considered.

## Introduction

Duchenne/Becker muscular dystrophy (DMD/BMD) is a progressive and irreversible muscle disease. It occurs in two types: the acute type, DMD, with an incidence of 1 in 3500–5000 live male births, and the milder type, BMD, which is five times less common (Emery 1991; Mendell and Lloyd-Puryear 2013; Moat et al. 2013; Romitti et al. 2015). DMD has an early onset (3–4 years of age), movement difficulties intensify rapidly and by the age of 9–14 years, patients become immobilised. Patients usually die in their late 20s or early 30s. The progression of BMD is slower and its signs and symptoms appear usually after 10 years of age. In many BMD patients, mobility is preserved for a long time. Sometimes, they start families, and their survival is often comparable to that in the general population (Emery 1991). Therapy of DMD based on exon skipping recently introduced is in development (Cirak et al. 2011).

Muscular dystrophies are caused by X-linked recessive mutations of the dystrophin gene, which is located in band 21 of the short arm of chromosome X (Hoffman et al. 1987). Most mutations result in a total loss of the protein product, dystrophin (DMD type), whereas some mutations, resulting in an in-frame deletion or insertion of three-nucleotide repetitions, allow for the synthesis of a truncated dystrophin molecule with a partially preserved function (BMD type).

The aim of the study was to present a group of families and individuals with a subclinical or very mild form of BMD, all with a very infrequently reported deletion of a single exon 48 of the dystrophin gene. The only indication for diagnostic tests was markedly elevated serum creatine phosphokinase (sCPK) (Table 1), which was usually an incidental finding. Most adult male carriers of this mutation do not consider themselves ill or disabled.

**Table 1:** Correlation of clinical findings in 17 males with exon 48 deletion from nine pedigrees

Patient/pedigree no.	Age (years)	Hypertrophy of the calves	Muscle aches	Post-exercise cramps	Increased fatigability	Decreased muscle tone	Decreased tendon reflexes	Decreased muscle force (mildly)	EMG^a^	sCPK^b^
1/I P	8	+	−	−	+	+	−	+	N	1891
2/II P	4	+	−	−	−	+	+	+	n.a.	5880
3/II	65	−	−	−	−	−	−	−	n.a.	n.a.
4/III P	8	−	+	−	+	−	+	−	Mild myopathic features^a^	4800
5/IV P	8	+	+	−	+	+	−	−	n.a.	3184
6/IV	36	+	−	+	−	−	−	−	n.a.	n.a.
7/IV	26	+	−	+	+	−	−	−	N	4400
8/V P	14	+	+	−	+	−	−	−	Mild myopathic features^a^	2800
9/V	68	+	−	−	+	−	+	−	n.a.	510
10/VI	4	−	−	−	−	+	+	+	N	1750 4730
11/VI P	9	−	−	−	+	+	+	+	N	978 9940
12/VI	65	−	−	−	−	−	−	−	n.a.	n.a.
13/VII P	5	+	+	−	+	+	−	+	N	14,723
14/VII	5	+	−	−	−	+	−	+	N	4895
15/VIII P	6	+	−	−	−	+	+	+	n.a.	2351 7382
16/IX P	6	−	−	−	+	+	−	?	N	902 2600
17/IX	2	+	−	−	?	+	-	?	N	2498 2656

*P* proband, *N* normal, *n.a.* not assessed

^a^EMG mild myopathic features, such as decreased amplitude of motor units and polyphasic records; examined muscles usually: m. biceps brachii, m. vastus, m. tibialis anterior, m. gastrocnaemius

^b^sCPK normal level 0–170 u

## Materials and methods

### Patients

Nine hundred and ninety unrelated cases of DMD/BMD were diagnosed based on molecular analysis, including 819 deletions, 90 duplications and 105 small mutations. In this group, there were 227 unrelated cases of BMD (23%). Among them, there were nine non-consanguineous families, in whom a deletion of exon 48 was detected. Genetic testing was carried out because of the elevated sCPK.

### DNA analysis

DNA was isolated from peripheral blood using the MagNA Pure Compact Instrument (Roche Instrument Center AG, Rotkreuz, Switzerland). The reagents used and the reaction itself were performed according to the procedure recommended by the manufacturer. The search for dystrophin gene mutations was performed using multiplex ligation-dependent probe amplification (MLPA), with the use of two kits of reagents (SALSA P034 and SALSA P035, MRC-Holland, Amsterdam, the Netherlands), which helped to assess all 79 exons of the dystrophin gene (http://www.mlpa.com). Reaction products were split by capillary electrophoresis (3130 Genetic Analyzer, Applied Biosystems, Waltham, MA, USA). Cases of single deletions (possible false results) were verified by multiplex polymerase chain reaction (PCR) analysis. In order to exclude consanguinity of nine families, haplotype analysis of three microsatellite loci in introns 45, 48 and 50 was performed (Clemens et al. 1991). Parents of the children and adult persons whose DNA was analysed signed the informed consent.

## Results

**Fig. 1:**
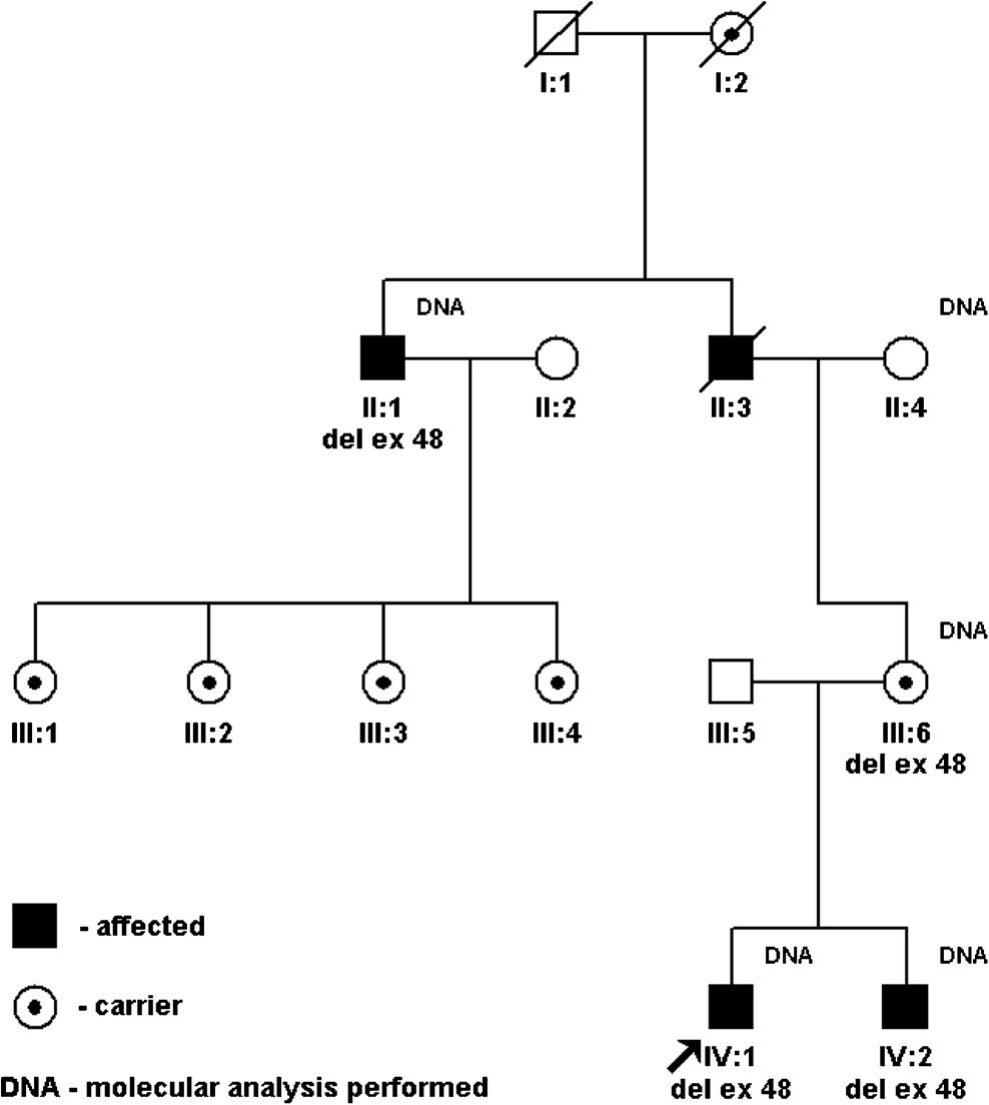
Pedigree VI (see Table 1). II:3 obligate male carrier of ex 48 del died at age 55 years of dilated cardiomyopathy

In-frame deletions of exon 48 of the dystrophin gene were detected in 17 males from the nine non-consanguineous families. The frequency of that deletion in the analysed material was 0.91% among all probands with molecularly detected mutation in the dystrophin gene (9/990) and 3.96% among probands with molecularly confirmed BMD (9/227). All cases of exon 48 deletion were familial. Apart from 17 males, the carrier status was confirmed in a total of 28 women (mothers, sisters, grandmothers, aunts). In four families, the deletion of exon 48 originated from maternal grandfathers. In families II and V (Table 1), examined grandfathers had the deletion. In families III and VI, the deceased, unexamined maternal grandfathers were also likely to have been carriers. This was indicated by their haplotype analysis and by the history data: one of the grandfathers had a characteristic calf hypertrophy, the second one—an obligate male carrier—died of dilated cardiomyopathy (Fig. 1). In two pedigrees, the familial character of the mutation was proven by finding a carrier status of the grandmothers. In the other three pedigrees, the probands’ mothers were carriers.

We did not find any indication of relationships between the analysed pedigrees.

Seventeen male patients with exon 48 deletion were evaluated clinically: the results are presented in Table 1. Those examined had elevated sCPK. Only four out of the 17 individuals reported lower extremities myalgia after significant exertion. Most physical examinations revealed calf hypertrophy of various degrees (11/17). There were no data indicating cardiomyopathy in the subjects with the deletion confirmed molecularly. The neurological examination in some of the patients revealed decreased muscle tone (10/17) and/or muscle weakness of very mild degree (9/17) and/or weakened tendon reflexes in the lower extremities (6/17). Adults did not reveal any significant neurological abnormalities; they had good exercise tolerance. Electromyography (EMG) was performed in ten individuals; only in two of them did the EMG graphs show mild myopathic features.

## Discussion

The possibility of asymptomatic deletions in the dystrophin gene was first postulated by Koenig et al., who suggested that such deletions can involve exons 31–44, without affecting the reading frame (Koenig et al. 1989). This hypothesis was confirmed by many authors who found that some mutations, usually in-frame deletions, are compatible with very mild or asymptomatic dystrophinopathy often identified because of an accidental finding of increased sCPK level. There are many examples of such reports: deletions of exons 17–48 (England et al. 1990); 1, 35–44, 45–48, 48–51, 51 (Beggs et al. 1991); 47 (Nordenskjöld et al. 1990); 45–51 (Saengpattrachai et al. 2006); 45–55 (Ferreiro et al. 2009); 10–27 (Dobosiewicz et al. 2000).

In this study in 17 males and 28 females from nine families, we detected an in-frame deletion of exon 48 within the dystrophin gene. According to the recommendation of MRC-Holland, the results were confirmed by multiplex PCR because a single exon deletion can be a false-positive by point mutation or polymorphism, changing the nucleotide sequence detected only by the MLPA probes. This mutation results in a dystrophin molecule shortened by 62 amino acids (rod domain region) but still containing intact, crucial regions responsible for binding to the glycoprotein complex proteins (Campbell and Kahl 1989). Deletion of exon 48 in the dystrophin gene based on the available literature seems to be very rare. As mentioned above, we found it in 9 out of 227 probands with BMD (3.96%), whereas the most extensive database on dystrophin gene mutations (Leiden University, http://www.dmd.nl) does not contain any case of a single exon 48 deletion. In the available literature, we found only seven reports on a single exon 48 deletion (Table 2). These include one case of 48 deletion in a group of 58 mild BMD patients (1.72%) (Beggs et al. 1991), two 5- and 10-year-old asymptomatic boys (Comi et al. 1994) and an American family with four asymptomatic males (the oldest one 58 years old) (Morrone et al. 1997). In Switzerland, there were two 9-year-old monozygotic twin brothers suffering from cramps and myalgia (Ramelli et al. 2006). In 2009, a French meta-analysis of 2084 patients with DMD/BMD mentioned 16 BMD patients with the deletion of exon 48 (0.77%), but there was only one report available of a 9-year-old boy who was asymptomatic apart from his elevated sCPK. No information on the clinical condition of the remaining patients was provided. However, the DMD Mutations Database edited by Montpellier University lists them as affected with BMD (Tuffery-Giraud et al. 2009). At the April 2011 Mediterranean Society of Myology Congress, 16 patients with BMD, aged 7–52 years, with the deletion of exon 48, were presented. The authors claimed that the patients had no symptoms of cardiomyopathy; however, no other data were provided on their clinical condition or consanguinity (Taglia et al. 2011). The above literature data are generally consistent with our observations. A clinical analysis of our patients, 17 male carriers of the mutation, 12 children (including nine probands) aged 2 to 14 years and five adults aged 26 to 68 years (all secondary cases), revealed in some of them only muscle hypotonia (10/17), mild muscle weakness (9/17) and decreased tendon reflexes (6/17). Adults, apart from very mild (rather unnoticed) muscle weakness and calf hypertrophy in some, had good exercise tolerance and no significant abnormalities on neurological assessment. Among our 17 patients, we did not find any case with evidence of cardiomyopathy. However, as mentioned above, according to the histories, there were two subjects not diagnosed molecularly, who died of: (1) dilated cardiomyopathy and (2) unspecified heart disease. Their positions in the pedigrees could suggest that they were carriers of the mutation (deletion of exon 48) (Fig. 1). Twelve of our patients were children (mean age 6.6 years). Therefore, one cannot be certain whether or not they will escape cardiomyopathy in future.

**Table 2:** Cases of muscle dystrophy with deletions of exon 48: data from the literature and own material

Source	Number of families	Number of cases	Age (years)	sCPK increased	Myogenic features (EMG)	Size of BMD group	% of BMD and comments
Beggs et al. (1991)	1	1	7	+	n.a.	58	1.72
Comi et al. (1994)	2	2	5, 10	+	n.a.	59	3.39
Morrone et al. (1997)	1	4	8, 28, 51, 58	+	n.a.	?	–
Ramelli et al. (2006)	1	2	9, 9	+	+	?	Monozygotic twins
Tuffery-Giraud et al. (2009)	10	16	?	+	n.a.	561	1.78
Taglia et al. (2011)	?	16	7–52	+	n.a.	?	–
Diegoli et al. (2011)	3	3	25, 38, 39	+	n.a.	34^a^	All three cardiomyopathy^a^
Own material	9	17	4–68	+	See Table 1	227	3.96

*n.a.* not assessed

^a^Three cases of exon 48 deletion out of 34 cases of dilated cardiomyopathy in whom mutations in the dystrophin gene were found

In 2011, three Italian patients with the deletion of exon 48 were described, diagnosed at the ages of 25, 38 and 39 years, who had severe cardiologic disorders; the two older ones died of congestive heart failure and the youngest one had heart transplantation (Table 2) (Diegoli et al. 2011). As our data may suggest, it is possible that the occurrence of very mild or almost asymptomatic cases of BMD may be considerably more frequent than generally believed, since many or most of them escape detection. Parents of the children carriers of exon 48 deletion are usually unaware of their children being affected, and possibly at risk of developing life threatening cardiomyopathy. The same problem may concern the adult carriers.

The detection of affected individuals should be carried on screening by cascade of relatives of the probands. Newborns screening programme, based on elevated sCPK level, may also be considered, such as the best known Welsh programme (Moat et al. 2013). Regular cardiologic assessments of the patients, including those who are ‘asymptomatic’ or revealing very mild phenotype, should be recommended (Hoogerwaard et al. 1999; Bastianutto et al. 2001).

Prenatal diagnostics remains an open issue. In families with mutations causing very mild BMD phenotype, including those with exon 48 deletion, pre-implantation genetic diagnostics can be offered.
